# Clinical Possibility of *Caenorhabditis elegans* as a Novel Evaluation Tool for Esophageal Cancer Patients Receiving Chemotherapy: A Prospective Study

**DOI:** 10.3390/cancers15153870

**Published:** 2023-07-29

**Authors:** Yuta Sato, Manabu Futamura, Yoshihiro Tanaka, Hiroshi Tsuchiya, Masahiro Fukada, Toshiya Higashi, Itaru Yasufuku, Ryuichi Asai, Jesse Yu Tajima, Shigeru Kiyama, Hideyuki Hatakeyama, Masayo Morishita, Takaaki Hirotsu, Eric di Luccio, Takuma Ishihara, Nobuhisa Matsuhashi, Kazuhiro Yoshida

**Affiliations:** 1 Department of Gastroenterological Surgery and Pediatric Surgery, Gifu Graduate School of Medicine, 1-1 Yanagido, Gifu 501-1194, Japan; 2 Department of Breast Surgery, Gifu University Hospital, 1-1 Yanagido, Gifu 501-1194, Japan; 3 Hirotsu Bio Science Inc., 22F The New Otani Garden Court, 4-1 Kioicho Chiyoda-ku, Tokyo 102-0094, Japan; 4 Innovative and Clinical Research Promotion Center, Gifu University Hospital, Gifu 501-1194, Japan

**Keywords:** esophageal cancer, *Caenorhabditis elegans*, N-NOSE, preoperative chemotherapy

## Abstract

**Simple Summary:**

Our research sought to elucidate the predictive capacity of Nematode Nose (N-NOSE) screening vis à vis the clinical implications of preoperative chemotherapy for patients suffering from esophageal cancer. This study focused on changes in the chemotaxis index of *Caenorhabditis elegans* before and after preoperative chemotherapy. When the target of the treatment effect was complete response only, the prediction accuracies calculated by area under the curve was 0.85 (95% Confidence interval: 0.62–1), and the sensitivity and specificity were 1 and 0.63, respectively. The results indicate that N-NOSE can accurately determine the response of the therapeutic effect of preoperative chemotherapy.

**Abstract:**

Background: The nematode *Caenorhabditis elegans* (*C. elegans*) possesses a sophisticated sense of smell and is used for a novel cancer screening test that utilizes the chemotaxis index. We designed a single-institution, prospective study to confirm the ability of Nematode Nose (N-NOSE) to determine preoperative chemotherapy’s efficacy for esophageal cancer patients. Patients and Methods: We investigated the predictability of N-NOSE screening for the clinical effects of preoperative chemotherapy for esophageal cancer patients receiving radical surgery. The index reduction score (IRS) was calculated via the chemotaxis of *C. elegans* at three points: before treatment, before surgery, and after surgery, and its clinical relevance was examined. Result: Thirty-nine patients with esophageal cancer were enrolled from August 2020 to December 2021, and 30 patients receiving radical surgery were examined. Complete response or partial response was achieved in 23 cases (76.7%). When the target of the treatment effect was complete response only, the prediction accuracies of the IRS calculated by area under the curve was 0.85 (95% Confidence interval: 0.62–1) in clinically achieving complete response group, and the sensitivity and specificity were 1 and 0.63, respectively. Conclusion: Index reduction score using N-NOSE screening may reflect the efficacy of chemotherapy for esophageal cancer patients. A large-scale prospective study at multiple centers is desired in the future.

## 1. Introduction

Worldwide, esophageal cancer is the fifth most common cause of cancer-related death for men and the eighth for women [1]. Esophageal cancer is relatively rare but still a significant global health concern. According to the World Health Organization, in 2020, there were an estimated 604,000 new cases of esophageal cancer worldwide and 544,000 deaths from the disease. Squamous cell carcinoma of the esophagus is also more common in eastern Asia and parts of Africa. Esophageal cancer is more common in men than women and tends to occur in people over 50. It is also more common in certain regions, such as eastern Asia and parts of Africa. The two main types of esophageal cancer are squamous cell carcinoma and adenocarcinoma. Squamous cell carcinoma is more common in developing countries and is often associated with tobacco and alcohol use. Adenocarcinoma is more common in developed countries and is often associated with obesity and gastroesophageal reflux disease (GERD).

The prognosis for esophageal cancer depends on various factors, such as the cancer stage and the individual’s overall health. However, early detection and treatment can improve outcomes. The American Cancer Society provides the following estimated 5-year relative survival rates for esophageal cancer based on stage. Localized (cancer is only in the esophagus): approximately 49% of people with localized esophageal cancer survive for at least 5 years after diagnosis; Regional (cancer has spread to nearby lymph nodes or tissues): approximately 26% of people with regional esophageal cancer survive for at least 5 years after diagnosis; Distant (cancer has spread to distant parts of the body): approximately 5% of people with distant esophageal cancer survive for at least 5 years after diagnosis.

Preoperative chemotherapy for locally advanced esophageal cancer is Japan’s current standard of care [2,3]. After controlling local and systemic micrometastases with preoperative chemotherapy, surgery has improved radicality and prolonged prognosis [4]. Still, esophageal cancer surgery is a highly invasive procedure, and depending on the underlying disease and physical status, there are populations in which surgery should be avoided. Complete histopathological response can be obtained through the use of a recent chemotherapy regimen [5]. Pathological response and prognosis may correlate, so determining preoperative chemotherapy’s efficacy is important [6].

Surgical options for esophageal cancer may include esophagectomy, endoscopic resection, and palliative surgery. Esophagectomy is the most common surgical procedure for esophageal cancer. The cancerous portion of the esophagus is removed, and the remaining portions of the esophagus and stomach are reconnected. This is a major surgical procedure involving incisions in the chest, abdomen, or neck and may require a hospital stay of several days to weeks. In contrast, endoscopic resection is a minimally invasive procedure that involves removing small, early-stage cancers from the lining of the esophagus using an endoscope (a flexible tube with a camera on the end). This procedure is generally only recommended for very early-stage cancers. Palliative surgery, however, is aimed at relieving symptoms, such as difficulty swallowing, and may involve inserting a stent to keep the esophagus open or bypassing the esophagus entirely.

Individuals who have an increased risk of developing esophageal cancer may undergo certain tests to detect the disease at an early stage. Endoscopy is the most common test to detect esophageal cancer. While endoscopy is generally considered safe, it does carry some risks, such as bleeding, infection, and perforation of the esophagus. Endoscopy to examine the lining of the esophagus for abnormalities is invasive and burdensome. Individuals with Barrett’s esophagus, a condition in which the cells lining the lower part of the esophagus have changed, may undergo regular endoscopies to check for signs of cancer. In addition to endoscopy, other imaging tests, such as computed tomography (CT) scans and positron emission tomography (PET) scans, may be used to detect esophageal cancer. It is important to note that screening tests for esophageal cancer are generally only recommended for individuals with a higher risk of developing the disease, such as those with a family history of the disease or a history of Barrett’s esophagus and a history of smoking and heavy alcohol use.

To improve survival rate and prognosis, the early detection of esophageal cancer must be improved. Esophageal cancer screening rates by country can vary widely, depending on several factors, including access to healthcare, screening policies, and cultural attitudes towards preventive care. Japan has one of the world’s highest rates of esophageal cancer screening, with 50% of Japanese adults undergoing esophageal cancer screening routinely. The United States has no formal screening program for esophageal cancer. However, some individuals may undergo screening as part of routine endoscopy procedures or if they are at high risk due to factors such as Barrett’s esophagus or a family history of esophageal cancer. In China, esophageal cancer is relatively common, and screening rates are increasing. The screening rate for esophageal cancer in China increased from 6.5% in 2008 to 21.1% in 2016. In Europe, there is no standardized esophageal cancer screening program. However, some countries, such as the Netherlands and the United Kingdom, have implemented targeted screening programs for high-risk individuals due to factors such as Barrett’s esophagus or a family history of esophageal cancer.

The development of cancer and cellular transformation can result in significant changes in both cellular and extracellular structures. The disease’s metabolic signatures have been detected in urine, with volatile organic compounds (VOCs) being one such pattern [7,8,9,10,11,12]. A range of cancers has been linked to variations in the levels of VOCs, which contribute to the distinctive “cancer smell” associated with the end product of metabolic changes. Dogs trained with olfactory associative learning can identify specific smells in urine and detect human lung and breast cancer instances.

Woollam et al. identified six VOCs via gas chromatography–mass spectrometry QTOF that could serve as the basis for a screening test with good separation of cancer/non-cancer (ROC AUC of 0.96) for breast cancer [13]. Colorectal cancer is the most frequent cancer in Western countries, and efforts are being made to detect it early. While screening and diagnosis rely on fecal occult blood tests, urine samples may be an excellent alternative, as they contain metabolome signatures and specific VOCs identified for colorectal cancer [14].

The nematode *C. elegans* possesses a sophisticated sense of smell that enables it to detect even slight changes in the concentration of odorants in its natural habitat and exhibit chemotaxis in the direction of bacteria, which serves as its food source. With approximately 1200 olfactory receptor-like genes (compared to around 800 in dogs), *C. elegans* approaches desirable odors while avoiding unpleasant ones [15]. Notably, research by Hirotsu et al. has shown that *C. elegans* is attracted to urine from cancer patients but tends to avoid urine from healthy individuals [16]. This response is primarily mediated through the AWA, AWB, and AWC sensory neurons responsible for detecting volatile odors. *C. elegans* exhibits a distinct chemotactic response toward the urine of an individual with cancer compared to that of a healthy individual [15,16,17,18]. Specifically, the AWA and AWC neurons elicit attractive behavior, while the AWB neuron mediates a repellent behavior [15].

The chemotaxis response of *C. elegans* to urine samples depends on the concentration of odorants [16]. These results provide the foundational support for the N-NOSE (Nematode Nose) multi-cancer primary screening test, which demonstrates a high degree of sensitivity in identifying cancer during its initial stages (85% average sensitivity stage O-I) [16,19,20,21]. N-NOSE is a novel cancer screening test that utilizes the chemotaxis index of *C. elegans*. It is commercially available in Japan and can detect the presence of 15 different types of cancer, including stomach, colorectal, lung, breast, pancreatic, liver, prostate, uterine, esophageal, gall bladder, bile duct, kidney, bladder, ovarian, and oral/pharyngeal cancers.

Although previous studies have compared nematode chemotaxis before and after surgery, no studies have examined whether N-NOSE can be used to determine the efficacy of preoperative or induction chemotherapy in the esophageal cancer patient. Thus, we designed a single-institution, prospective study to confirm the ability of N-NOSE as a tool to determine the efficacy of preoperative or induction chemotherapy for esophageal cancer patients.

## 2. Patients and Methods

### 2.1. Eligibility Criteria

The current study was executed with a cohort of patients who had undergone preoperative or induction chemotherapy and elective esophagectomy for thoracic esophageal cancer at the Department of Gastroenterological Surgery and Pediatric Surgery at Gifu University in Japan. The inclusion criteria specified patients to be older than 20 years at the point of registration and to possess a histologically certified diagnosis of Stage II-IVb esophageal squamous cell carcinoma.

Additional criteria for selection included an Eastern Cooperative Oncology Group performance status ranging from 0 to 1; a life expectancy exceeding 12 weeks; and adequate liver, bone marrow, renal, and cardiovascular functions (parameters included serum bilirubin ≤ 1.5 mg/dL; neutrophil count ≥ 1500/mm^3^; serum aspartate aminotransferase and alanine aminotransferase levels ≤ twice the upper limit of the standard range; platelet count ≥ 10 × 10^4^/mm^3^; hemoglobin ≥ 8.0 g/dL; creatinine ≤ 1.2 mg/dL [or creatinine clearance ≥ 30 mL/min], and platelet count > 10 × 10^4^/L).

Patients with serious simultaneous illnesses, symptomatic infectious diseases, severe allergies, peripheral neuropathy, and uncontrolled diabetes mellitus were excluded from the study. Those who met the inclusion standards were registered successively among other esophageal cancer patients within the timeframe of August 2020 to December 2021.

The next phase of the study pertained to the determination of the treatment approach and the assessment of the chemotherapy’s efficacy.

### 2.2. Treatment Strategy and Evaluation of Chemotherapy

**Figure 1:**
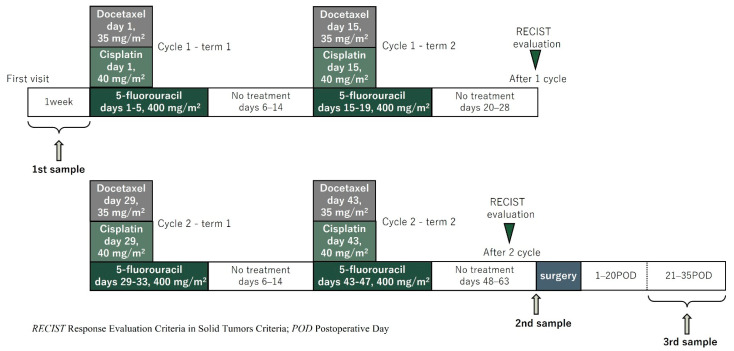
Preoperative or induction chemotherapy regimens and timing of urine sampling collection.

Patients received TXT diluted in 250 mL of normal saline at a dose of 35 mg/m^2^. CDDP was then prepared in normal saline at a dose of 40 mg/m^2^ and administered intravenously (IV) over 2 h on day 1. 5-FU was prepared in normal saline at a dose of 400 mg/m^2^ and administered IV continuously on days 1–5. TXT and CDDP were given on days 1 and 15, and 5-FU was given on days 1–5 and 15–19 of every 28-day cycle (one cycle). All included patients were scheduled to receive two cycles [5,22,23] (Figure 1). Disease staging was defined according to the International Union Against Cancer TNM classification system, 8th edition [24]. Measurable lesions were evaluated with computed tomography or magnetic resonance imaging, except for the primary tumor. They were assessed in accordance with the Response Evaluation Criteria in Solid Tumors Criteria (RECIST) version 1.1 [25].

### 2.3. Study Endpoints

We investigated the predictability of N-NOSE screening for the clinical effects of preoperative chemotherapy for esophageal cancer patients receiving radical surgery.

### 2.4. Measurement Method of N-NOSE and Index Reduction Scores

**Figure 2:**
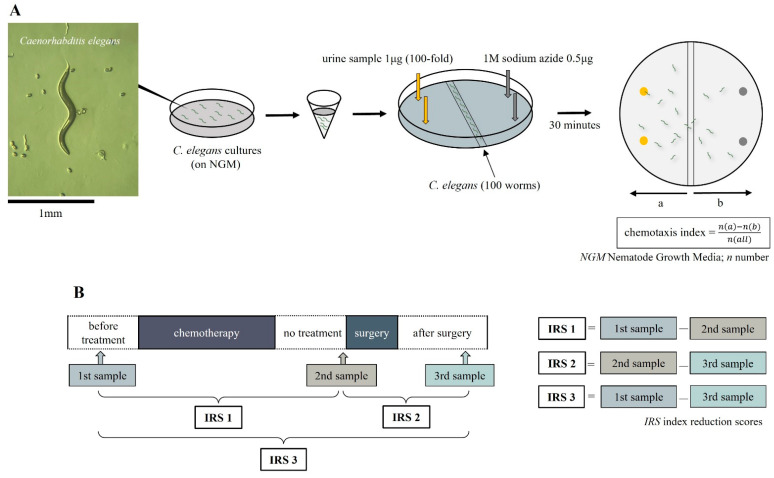
(**A**) Measurement method of *Caenorhabditis elegans* chemotaxis index. (**B**) The difference in the chemotaxis index obtained from each therapeutic point was defined as the index reduction score.

Urine samples were collected from enrolled esophageal cancer patients at three time points: before the start of chemotherapy, after the end of chemotherapy, and after surgery at each. The first was taken within a week before the start of chemotherapy. The second collection was taken the day before or the morning of surgery to lessen the effect of anticancer drugs on the chemotaxis index. The third was taken 3 to 5 weeks after surgery (Figure 1). The gathered urine specimens were promptly frozen at or below −20 °C within two hours post-collection and remained in this frozen state until their analysis using N-NOSE. *C. elegans* (wild-type N2) were cultivated using conventional methodologies at a controlled temperature of 20 °C. They were grown on Nematode Growth Media (NGM), seeded with the Escherichia coli strain NA22, serving as a food source. [16,26]. Chemotaxis assays were performed on 9 cm plates containing 10 mL of 2% agar, 5 mM KPO_4_, 1 mM CaCl_2_, and 1 mM MgSO_4_, as previously described [16]. The nematode chemotaxis analysis was conducted using standard protocols [16]. Briefly, 0.5 μL of 1 M sodium azide was added to two points at both ends of the plates, and then 1 μL of urine sample diluted to 100-fold with ultra-pure water was added to only two points on the side of the plate. The purpose of sodium azide is to minimize the effects of adaptation, and it was added before adding the worms to the plate. Sodium azide was added to four points of the plate (urine side and non-urine side). Roughly 100 synchronized young adult worms were collected and washed three times with chemotaxis buffer (0.05% gelatin, 5 mM KPO_4_, 1 mM CaCl_2_, and 1 mM MgSO_4_) placed in the center of the plate. After excess buffer removal, the worms were allowed to roam for 30 min. The chemotaxis index was calculated by dividing the number of worms near the urine sample and the number of worms in the region without the sample by the total number of worms [17,27]. The chemotaxis index was determined for each urine sample at 100-fold dilution for chemotaxis assay. A positive index (0 to 1) indicates an attraction to the sample, while a negative index (−1 to 0) indicates repulsion to the sample (Figure 2A). In this study, we focused on the difference in the chemotaxis index. The difference in the chemotaxis index obtained from each therapeutic point was defined as the index reduction score (IRS). In other words, the difference before treatment (1st sample) and before surgery (2nd sample) was defined as IRS1, and the difference before surgery (2nd sample) and after surgery (3rd sample) was defined as IRS2. The difference before treatment (1st sample) and after surgery (3rd sample) was defined as IRS3 and its clinical relevance was examined (Figure 2B). The Chemotaxis index was calculated by several technicians who were not informed of any antitumor effect.

### 2.5. Statistical Analyses

Statistical analyses, including the Wilcoxon rank-sum test (Mann–Whitney test), One-way ANOVA, and Receiver Operating Characteristic (ROC) analysis to calculate the area under the curve (AUC) values, were conducted using JMP^®^ 14 (SAS Institute, Cary, NC, USA). Statistical significance was defined as a *p*-value of less than 0.05.

### 2.6. Ethics Approval

This study was conducted in accordance with the World Medical Association Declaration of Helsinki. This study protocol was approved by ethics committees of Gifu University School of Medicine (ID: 2022-0107), and written informed consent was obtained from all the patients before all study-related procedures.

## 3. Results

**Figure 3:**
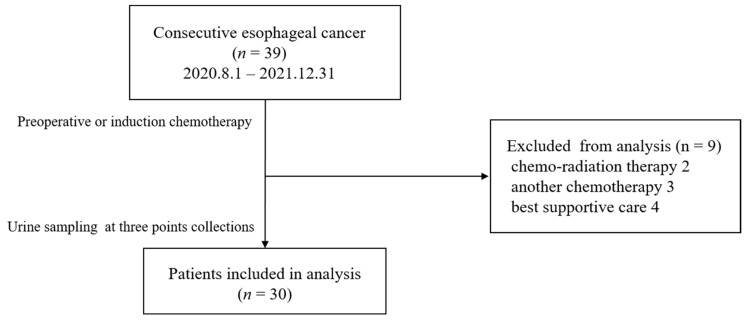
Study exclusion criteria.

**Figure 4:**
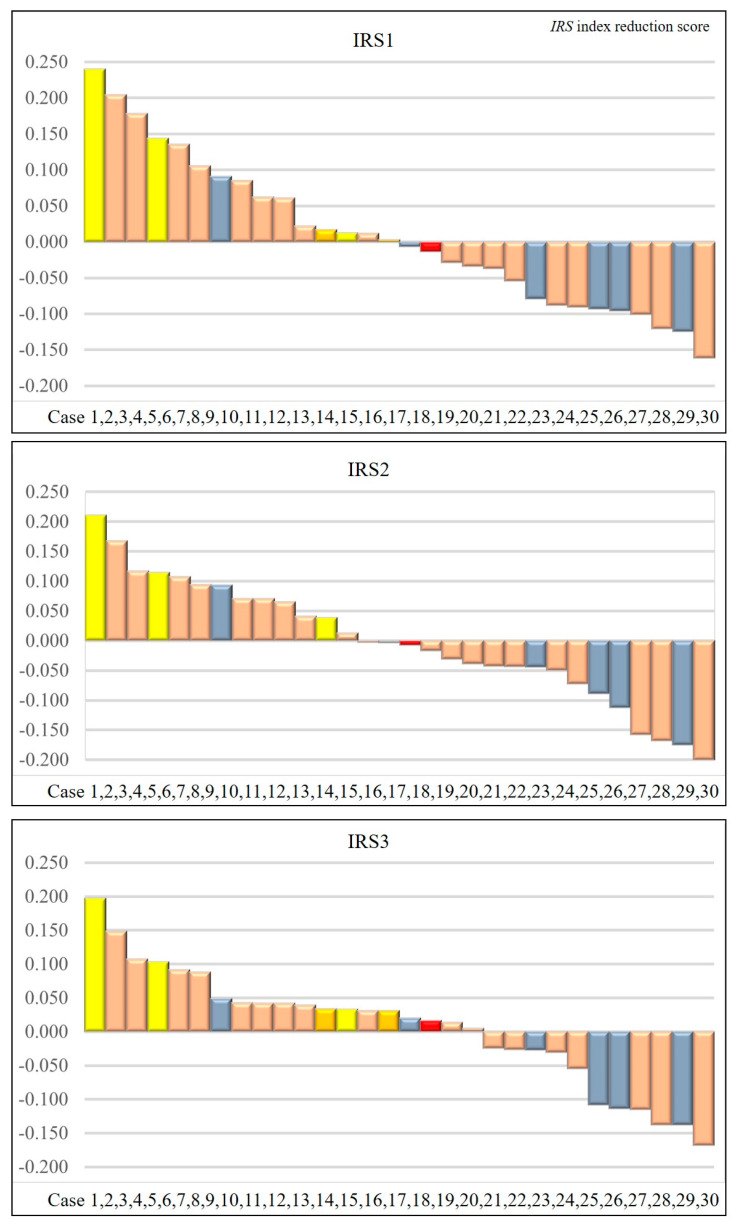
Index reduction score (IRS)1, IRS2, and IRS3 values for the 30 cases analyzed. Complete response: yellow; Partial response: pink; Stable disease: gray; Progression disease: red.

Thirty-nine patients with esophageal cancer were enrolled from August 2020 to December 2021. Preoperative or induction chemotherapy was introduced for all patients on the assumption of radical resection. Nine patients could not undergo surgery due to side effects of chemotherapy, exacerbation of comorbidities, or refusal of treatment. Of the nine patients, two received chemoradiotherapy, three received another chemotherapy, and four were discontinued (Figure 3). Finally, we analyzed 30 patients. The age, sex, TNM classification, and clinical response are summarized in Table 1. The median age was 72 years old. Complete response (CR) or partial response (PR) was achieved in 23 cases (76.7%). Figure 4 shows the IRS1, IRS2, and IRS3 values and efficacy for the 30 cases analyzed.

When comparing the response group (CR or PR) and the non-response group (SD or PD), IRS1s were 0.003 [−0.055, 0.091]: median [interquartile range], and −0.088 [−0.101, −0.061], respectively, with a *p*-value of 0.288. IRS2s were −0.003 [−0.044, 0.071] and −0.039 [−0.158, 0.107], respectively, with a *p*-value of 0.598. IRS3s were 0.033 [−0.026, 0.088] and −0.027 [−0.114, −0.020], respectively, with a *p*-value of 0.107 (Table 2). For the treatment effect for CR, IRS1 was 0.135 [0.003, 0.240], IRS2 was 0.094 [−0.003, 0.167], and IRS3 was 0.103 [0.033, 0.197].

**Figure 5:**
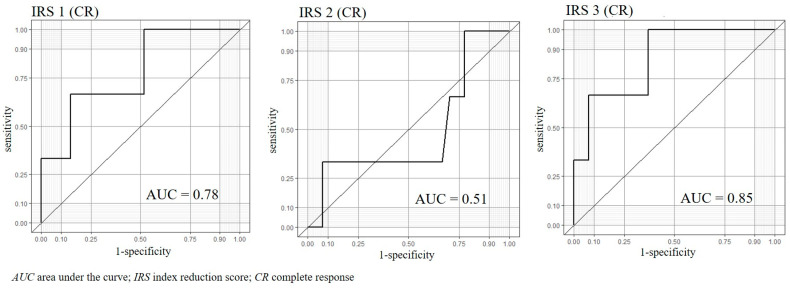
The area under the curve of index reduction score when the target of treatment effect is a complete response.

The prediction accuracies of the three IRSs obtained from 30 patients are summarized in Table 3. For the treatment effect of CR or PR, the three IRSs were IRS1: AUC = 0.64 (95% confidence interval (CI): 0.36–0.91), IRS2: AUC = 0.57 (95% CI: 0.28–0.86), and IRS3: AUC = 0.7 (95% CI: 0.49–0.92), respectively. On the other hand, when the target of the treatment effect was CR only, the three IRSs were IRS1: AUC = 0.78 (95% CI: 0.46–1), IRS2: AUC = 0.51 (95%CI: 0.06–0.96), and IRS3: AUC = 0.85 (95% CI: 0.62–1), respectively (Figure 5).

## 4. Discussion

The chemotaxis response of *C. elegans* to urine samples depends on the concentration of the odorant [16]. These findings are the basis for the N-NOSE multi-cancer primary screening test, which is highly sensitive in detecting cancer at its early stages (85% average sensitivity stage 0-I) [16,20,21]. Thus, N-NOSE, commercially available in Japan since late 2020, serves as a primary screening test for cancer. It is characterized by a minimally invasive and simple test using the patient’s urine, yet it has a very high sensitivity and specificity, screening even early-stage cancers. Very few clinical references report the relationship between the N-NOSE chemotaxis index and esophageal cancer. Only one patient in the Hirotsu et al. study [16], one patient in the Inaba et al. study [21], and 18 esophageal cancer patients in the Kusumoto et al. study [19] were included. So, evaluations on N-NOSE, to date, focused solely on esophageal cancer patients are scarce. Moreover, only a few studies have focused on changes in the N-NOSE index before and after surgery, i.e., before and after tumor resection. Kusumoto et al. performed a clinical study with the aim of assessing the efficacy of N-NOSE as a postoperative instrument for detecting the presence of both gastric and colorectal cancers. The study demonstrated, via ROC analysis, that N-NOSE outperformed both CEA and CA19-9 in its accuracy correlating with the removal of malignant tumors [19]. The findings implied that the changes in urinary cancer-specific odors triggered by surgical removal were mirrored in the olfactory behaviors of *C. elegans*. Similarly, Asai et al. carried out a comparative analysis of the chemotaxis index in 83 patients suffering from pancreatic cancer, both pre- and post-surgery [20]. Preoperative urine samples had a significantly higher chemotaxis index than postoperative samples in patients with pancreatic cancer, with an AUC = 0.845 (10^−1^ dilution of urine) and AUC = 0.820 (10^−2^ dilution). Additionally, this approach, which utilizes alterations in the chemotaxis index from the preoperative to the postoperative phase, exhibited superior sensitivity in identifying early-stage pancreatic cancer compared to traditional diagnostic markers, namely CEA and CA19-9 [20]. Ferrari et al. reported that the pattern of urinary chemical components, including volatile organic compounds in patients with cancer, changed following surgical therapy, and it is considered that such changes are detected by *C. elegans* [28].

We focused on changes in the chemotaxis index before and after preoperative chemotherapy for esophageal cancer. The novelty of this study lies in its focus on the relationship between the shrinking effect of anticancer drugs and changes in the olfactory behaviors of *C. elegans*. We used the IRS, which might reflect chemotherapy’s biological effect on cancer cells in vivo in the patients after chemotherapy. The results did not show significant AUC for all three IRSs for CR or PR treatment effect. On the other hand, when the treatment effect was limited to CR only, the AUC values were higher in IRS1 and IRS3 (AUC = 0.78 and IRS3: AUC = 0.85, respectively). The low AUC in the study, including CR and PR cases, may reflect that the nematodes reacted to the residual esophageal cancer in the PR cases. The result may support the screening ability of N-NOSE, which can detect even early-stage cancers. In the group in which CR was obtained, the presence of cancer disappeared on the image, and the AUC of IRS2 was relatively low. In addition, the AUC of IRS1 and IRS3 were relatively high. This result indicates that N-NOSE can accurately determine the response of the therapeutic effect of preoperative chemotherapy, which is confirmed by the fact that the cases achieved CR with preoperative treatment had a low change in *C. elegans* chemotaxis after surgery.

Recently, new methods for determining the presence of biological cancer cells have been developed. For example, liquid biopsy in rectal cancer has been reported to monitor response to chemoradiation therapy and assess the risk of disease recurrence [29]. On the other hand, there is also a report that squamous-cell-carcinoma-related antigen, which is a classical tumor marker, does not reflect the disease activity of esophageal cancer [30]. Our prospective study using *C. elegans* suggested that the non-invasiveness of N-NOSE may be comparable to the usefulness of biological cancer diagnosis, such as liquid biopsy.

This study has several limitations. First, the number of patients enrolled remains insufficient. Second, it is unclear whether the factor that affected the chemotaxis index of the nematodes was simply tumor shrinkage alone. The possibility that the drugs used for chemotherapy or the patient’s general condition affected the nematode’s movement cannot be ruled out. It is important to note that this study focused on evaluating the changes in the chemotaxis index of *C. elegans* before and after preoperative chemotherapy, and as such, no control was included. In addition, since N-NOSE is a tool for assessing the presence of cancer, setting a cut-off value and focusing on the changes in the chemotaxis index requires further investigation.

## 5. Conclusions

Index reduction score using N-NOSE screening may reflect the efficacy of chemotherapy for esophageal cancer patients. A large-scale prospective study at multiple centers is desired in the future.

## Figures and Tables

**Table 1:** Baseline characteristics of the patients.

Characteristics	Number of Patients (*n* = 30)
Age (median, range)	72 (61–82)
Sex (Male/Female)	24/6
clinical T stage (2/3/4b)	2/16/12
clinical N stage (0/1/2/3)	2/17/7/4
clinical M stage (0/1)	28/2
Stage (II /III/IVa/IVb)	3/12/10/5
CR/PR/SD/PD	3/20/6/1

*CR* complete response; *PR* partial response; *SD* stable disease; *PD* progression disease.

**Table 2:** Comparison of response group (CR or PR) and non-response group (SD or PD).

	CR or PR (*n* = 23)	SD or PD (*n* = 7)	*p*-Value
IRSI	0.003	−0.088	0.288
	[−0.055, 0.091]	[−0.101, −0.061]	
IRS2	−0.003	−0.039	0.598
	[−0.044, 0.071]	[−0.158, 0.107]	
IRS3	0.033	−0.027	0.107
	[−0.026, 0.088]	[−0.114, −0.02]	

*CR* complete response; *PR* partial response; *SD* stable disease; *PD* progression disease; *IRS* index reduction score.

**Table 3:** Predictive accuracy of three IRSs for 30 patients.

	CR					CR or PR				
	AUC	95%CI	Sensitivity	Specificity	Cutoff Point	AUC	95%CI	Sensitivity	Specificity	Cutoff Point
IRS 1	0.78	0.46–1	0.67	0.85	0.1	0.64	0.36–0.91	0.83	0.57	−0.08
IRS 2	0.51	0.06–0.96	0.67	0.67	−0.04	0.57	0.28–0.86	0.57	0.71	−0.01
IRS 3	0.85	0.62–1	1	0.63	0.03	0.7	0.49–0.92	0.61	0.86	0.03

*IRS* index reduction score; *AUC* area under the curve; *CI* confidence interval; *CR* complete response; *PR* partial response.

## Data Availability

Data availability is restricted due to privacy and ethical restrictions.
